# The relationship between mindfulness, psychological flexibility, and symptom severity in persons with schizophrenia-spectrum-disorders: a cross-sectional study

**DOI:** 10.1038/s41598-025-03380-2

**Published:** 2025-06-04

**Authors:** Inge Hahne, Julia Segerer, Marco Zierhut, Niklas Bergmann, Thi Minh Tam Ta, Eric Hahn, Kerem Böge

**Affiliations:** 1 Department of Psychiatry and Neurosciences, Campus Benjamin Franklin, Charité – Universitätsmedizin Berlin, Berlin, 12203 Germany; 2 German Center of Mental Health (DZPG), Berlin, Germany; 3 Department of Psychology, Ludwig-Maximilians-Universität München (LMU), Munich, Germany; 4 Medical University Brandenburg, Neuruppin, Germany; 5 Department of Education and Psychology, Clinical Child and Adolescent Psychology and Psychotherapy, Freie Universität Berlin, Berlin, Germany

**Keywords:** Schizophrenia spectrum disorders, Mindfulness, Psychological flexibility, Mediation, Negative symptoms, Depressive symptoms, Human behaviour, Schizophrenia

## Abstract

The effectiveness of mindfulness-based interventions (MBIs) in enhancing mental well-being and reducing positive, negative, and depressive symptoms in schizophrenia spectrum disorders (SSD) has been increasingly supported by evidence. However, the underlying mechanisms of MBIs require further examination. Psychological flexibility (PF), typically assessed through cognitive fusion, has been associated with clinical change in MBIs. This study employed a cross-sectional design to explore the interplay between mindfulness, PF, and symptom severity in SSD. A total of *N* = 94 persons with SSD were included in the analysis. Correlation and mediation analyses were performed using PROCESS analysis, with positive, negative, and depressive symptom severity as outcome variables, measured by the Positive and Negative Syndrome Scale (PANSS) and the Depression Anxiety Stress Scale (DASS-21), respectively. The findings indicated that mindfulness was significantly negatively correlated with positive and depressive symptoms and significantly positively correlated with PF. A significant mediating effect of PF was identified in the relationship between mindfulness and both negative and depressive symptoms. This study supports previous research suggesting PF as a possible mechanism of action in MBIs. However, future research utilizing longitudinal designs, more nuanced analyses, and mixed method approaches in assessment tools is warranted.

## Introduction

Mindfulness has emerged as a promising approach for improving mental health in recent years. It involves the process of consciously experiencing the current state of one’s body, mind, and environment without being distracted by inner thoughts, memories, fantasies, or strong emotions, and without evaluating these perceptions^1^. Mindfulness-based treatment approaches have been found effective in addressing various conditions, including chronic pain^2,3^, substance misuse^4^, depressive symptoms^5^, as well as anxiety and stress^6^. However, reports of adverse events, such as depersonalization following intensive meditation, raised concerns about the potential negative effects of mindfulness practices on persons with schizophrenia spectrum disorders (SSD)^7^. As a result, there was initial hesitancy in undertaking research for its use in SSD, particularly in comparison to research on more common mental disorders like anxiety and depression^8^. Nevertheless, adaptations such as small group delivery, shorter sessions, avoidance of prolonged silence, and the use of simple language have rendered mindfulness practices safe for SSD patients^8^. Moreover, mindfulness-based interventions (MBIs) are (cost-) effective and accessible, especially when delivered in groups^9,10^.

Evidence from meta-analyses on the effectiveness of mindfulness- and acceptance-based interventions for improving symptom severity in SSD is promising but mixed^10–15^. Some meta-analyses^11,12,14,15^ report significant small effects on positive symptoms (Hedge’s *g* = 0.32–0.48), while others did not find these effects^10,13^. Similarly, significant effects on negative symptoms (*g* = 0.24–0.75) were identified in a subset of meta-analyses^12–14^, but not in others^10,11^. A recent systematic meta-review^16^ including 18 meta-analyses reported promising effects of MBIs on negative symptoms, with small to large effect sizes (*g* = 0.38–0.98), while only 50% of the included meta-analyses found significant effects for positive symptoms (*g* = 0.16–0.42).

Next to positive and negative symptoms, also depressive symptoms show high prevalence rates in SSD, with 29% of persons with SSD fulfilling the criteria for clinically significant comorbid depression^17^. Depressive symptoms in SSD are associated with poorer clinical outcomes and disease progression as well as with heightened suicide risk^18–20^, which makes early detection and treatment highly important. A study by Bergmann et al.^21^ found higher mindfulness in SSD to be associated with lower depressive symptoms, and two of the above-mentioned meta-analyses reported that mindfulness- and acceptance-based interventions are especially effective in treating depressive symptoms in SSD (*g* = 0.14–0.80)^10,13^. However, the overall evidence remains inconclusive, as some meta-analyses did not find significant effects on depressive symptoms^11,15^. In a recent systematic meta-review only 50% of the included meta-analyses found significant effects for affective symptoms (*g* = 0.28–0.97)^16^.

The discrepancies in these meta-analytic findings may be explained by differences in intervention components, as well as variations in treatment setting, duration, and instructor experience^22^. Taken together, even though there is a growing body of evidence depicting the relationship between mindfulness and symptom reduction in SSD, these results underline the importance of continuative research and further advances in this area.

Apart from the question of which specific symptoms change through mindfulness, the mechanisms behind these effects are poorly understood. Persons with SSD may benefit from MBIs by developing an altered relationship with their symptoms, fostering a more accepting attitude, and gaining the ability to disengage from distressing experiences such as hallucinations or delusions^23^. This shift could reduce symptom-related stress, as persons with SSD frequently become absorbed in their symptoms or engage in dysfunctional behaviours, such as avoidance strategies^11^. Shapiro et al.^24^ suggest that enhancing cognitive, emotional, and behavioural flexibility might be one mechanism through which mindfulness produces positive effects. In recent years, psychological flexibility (PF) has frequently been linked to clinical improvement within the mindfulness framework^25^. PF is defined as the ability to consciously engage with the present moment in an accepting way, guided by chosen values and long-term goals rather than by distressing internal experiences^26^. Compared to healthy individuals, persons with SSD exhibit lower PF^27–29^. Increasing PF is also the primary objective of acceptance and commitment therapy (ACT), one of the most widely used manualized approach in acceptance and mindfulness-orientated treatments^30,31^.

Research focusing on PF frequently employs the Cognitive Fusion Questionnaire (CFQ) as a primary assessment tool^32^. According to the theoretical framework of ACT, cognitive fusion (CF) is a central process in PF and a key element of psychopathology^30^. CF occurs when behaviour is overly regulated and influenced by cognition rather than external information, leading individuals to interpret their thoughts literally rather than recognizing them as transient states^29,32,33^. The process of distancing from these rigid thoughts is referred to as cognitive defusion^32^, a core target of many interventions based on ACT. In conclusion, individuals with high levels of CF tend to identify closely with their internal states and thoughts, resulting in reduced PF.

CF was found to be related to lower levels of mindfulness^34,35^ as well as to poor mental health outcomes such as depression and anxiety^32,36,37^. Furthermore, several studies indicate that CF mediates the relationship between mindfulness and negative affect^38,39^, symptoms of depression and anxiety in healthy individuals^37^ as well as negative symptoms^25,27^. For example, one of the first studies of its kind found that PF mediates the negative relationship between mindfulness and negative symptom severity in persons with SSD^25^.

The current body of research on this topic is limited, with only a small number of studies, some of which involved relatively small sample sizes, focusing on the interplay between symptom severity, mindfulness, and PF in persons with SSD. The present study first aims to examine the association between mindfulness and positive, negative, and depressive symptoms, given that existing evidence remains inconclusive. Additionally, it seeks to replicate the association between mindfulness and PF, as well as the mediating role of PF in the relationship between mindfulness and symptom severity. For the first time, depressive symptoms are incorporated into this model.

It was hypothesized that in persons with SSD (H1) mindfulness is negatively correlated with (H1a) positive symptoms, (H1b) negative symptoms, and (H1c) depressive symptoms. Furthermore, it was expected that (H2) mindfulness is positively correlated with PF; and lastly, that (H3) PF mediates the relationship between mindfulness and (H3a) positive symptoms, (H3b) negative symptoms as well as (H3c) depressive symptoms.

## Methods

### Design

**Fig. 1:**
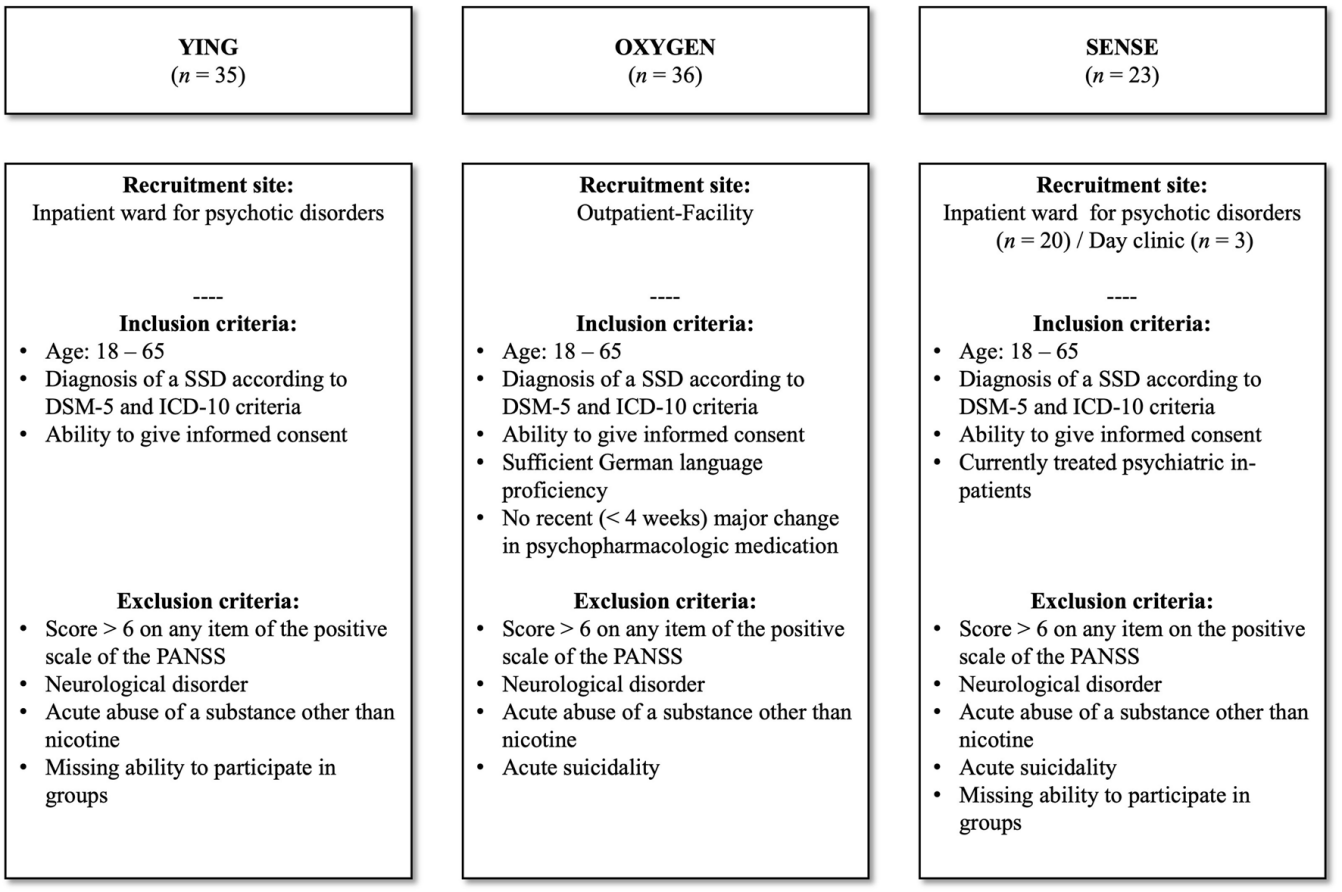
Data from participants (*N* = 94) at baseline from three separate trials at the Department of Psychiatry and Neurosciences at Charité—Universitätsmedizin Berlin, Campus Benjamin Franklin, Germany. *SSD* Schizophrenia Spectrum Disorders, *DSM-5* Diagnostic and Statistical Manual of Mental Disorders 5th edition, *ICD-10* International Classification of Diseases 10th edition, *PANSS* Positive and Negative Syndrome Scale.

A cross-sectional design was employed to accomplish the primary objectives of the study. The data were derived from baseline assessments across three trials conducted at the Department of Psychiatry and Neurosciences at Charité—Universitätsmedizin Berlin, Campus Benjamin Franklin, Germany, and were collected between 06/2018 and 05/2023. For additional details on these trials, refer to Fig. 1. The studies were conducted in accordance with the latest version of the Declaration of Helsinki and were approved by the ethical committee of the Charité—Universitätsmedizin Berlin (EA4/239/20, EA4/233/21, EA4/053/18), as well as pre-registered on clinicaltrials.gov (NCT04730518, NCT05491486, NCT03671005). Data collection was conducted using a tablet-based system via REDCap^40^, a secure, web-based software platform designed for research data collection and management. The data were securely and pseudonymously stored and processed.

### Participants and procedure

Participants from all three trials were recruited from the inpatient ward for psychotic disorders, the psychiatric day clinic as well as the outpatient facility of the Charité—Universitätsmedizin Berlin, Campus Benjamin Franklin, Germany. Patients who met the inclusion criteria for study participation (see Fig. 1) were identified by licensed psychiatrists, psychologists, or research assistants working in the respective clinical setting, and discussed in team meetings to assess eligibility. Subsequently, eligible patients were contacted by a research assistant, either in person or by telephone. Patients with severe positive symptoms, as indicated by a score of seven on any item on the Positive Syndrome Subscale of the Positive and Negative Syndrome Scale (PANSS), a neurological disorder or an acute substance dependence, apart from nicotine, were excluded from participation. For a detailed description of all exclusion criteria see Fig. 1. The participation in the study was entirely voluntary, and withdrawal was possible at any time.

After giving written informed consent, participants provided sociodemographic information and completed three self-rating questionnaires, followed by one rater-based assessment in German language. Rater were blinded, and consisted of licensed psychiatrists and psychologists. Ten patients participated in more than one of the three studies. In these cases, only the most recent data was used for analysis.

### Measures

#### Mindfulness

To measure mindfulness, the German version of the Southampton Mindfulness Questionnaire (SMQ) was administered^41^. It refers to the past seven days and conceptualizes mindfulness as four related aspects: (1) decentered awareness, (2) letting go, (3) non-judgment, and (4) non-aversion. The SMQ comprises 16 items that are rated on a seven-point Likert scale ranging from (6) “*agree totally*” to (0) “*disagree totally*”. Consequently, the total score ranges from 0 to 96, with a higher score indicating higher mindfulness. The internal consistency was good, with a Cronbach’s α = .89^41^. In this sample, a Cronbach’s α of .70 was found.

#### Symptom severity

For the assessment of the severity of positive and negative symptoms, the Positive and Negative Syndrome Scale (PANSS) was employed^42^. The PANSS assesses three symptom domains: positive symptoms, negative symptoms, and general psychopathology. It consists of 30 items that are rated on a seven-point Likert scale, with higher scores indicating greater symptom severity, ranging from (1) “*absent*” to (7) “*extreme*”. The positive scale and the negative scale consist of seven items each. The internal consistency was satisfactory, with Cronbach’s α = .62 for PANSS positive scale, α = .92 for PANSS negative scale, and α = .55 for PANSS general scale^43^. In this sample, the internal consistency for the PANSS positive scale was α = .68, and for the PANSS negative scale α = .74.

The depression subscale of the German version of the Depression Anxiety Stress Scale (DASS-21) was used to measure the severity of depressive symptoms in clinical and nonclinical samples^44^. This questionnaire consists of 21 items, with seven items representing the depression domain. Participants are asked to rate how much each statement applied to them over the past week on a 4-point Likert scale ranging from (0) “*did not apply to me at all*” to (3) “*applied to me very much or most of the time*”. The total score for each domain can range from 0 to 21, with higher scores indicating greater severity of symptoms. In clinical and nonclinical samples, depressive symptoms are considered either normal (0–9), mild (10–13), moderate (14–20), or severe (21). The German version of the DASS-21 has demonstrated excellent internal consistency (Cronbach’s α = .91) for the depression subscale^45^. For the current study, the reliability of the depression subscale was excellent as well (α = .85).

#### Psychological flexibility

To measure PF, the German version of the Cognitive Fusion Questionnaire (CFQ) was employed^46^. The questionnaire is a self-report measure with seven items that are scored on a 7-point Likert scale, ranging from (7) “*always true*” to (1) “*never true*”. The total score can range from 7 to 49, with higher scores indicating higher CF, which indicates lower PF. Previous research demonstrated that the CFQ has excellent internal consistency, with Cronbach’s α = .95^46^. The present study showed an α of .85.

### Statistical analysis

**Fig. 2:**
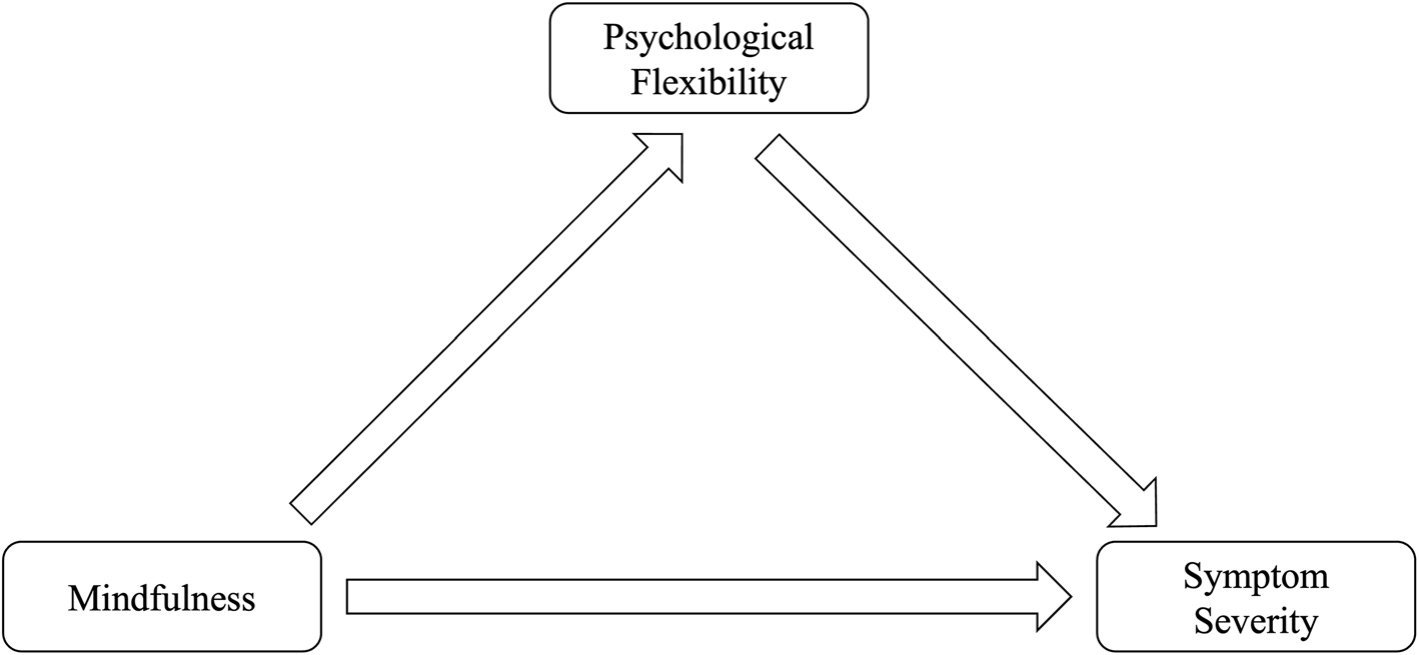
Overall model of the relationship between mindfulness, psychological flexibility, and symptom severity of positive symptoms (Model A), negative symptoms (Model B), and depressive symptoms (Model C).

Data were analyzed with the R program for Statistical Computing (version 4.2.3) for macOS^47^. First, zero-order correlation coefficients were calculated to determine whether the (H1) SMQ sum score is significantly related to the (H1a) PANSS positive scale sum score, (H1b) PANSS negative scale sum score, and (H1c) DASS-21 depression scale sum score, as well as to the (H2) CFQ sum score. For hypotheses 3a-c, atemporal mediation models were analyzed using the ordinary least squares regression method, implemented through PROCESS Model 4^48^. In these models, the sum scores for the PANSS positive scale (Model A), the PANSS negative scale (Model B) and the DASS-21 depression scale (Model C) were treated as the DV, with the SMQ sum score as the IV and the CFQ sum score as the mediator. An overview of the overall model is provided in Fig. 2*.*

To evaluate linearity, plots were visually inspected. Potential outliers in each of the three paths of the model (see Fig. 2) were identified using Cook’s distance, and participants with scores exceeding .03 were excluded from the analysis. Bootstrapping, a robust procedure that is independent of the distributional properties of the dataset^49^, with 10,000 estimates, was applied to compute inferential statistics and 95% confidence intervals for the effects, which were considered significant if zero was not included. In all analyses, a significance level of α = .05 was applied.

Post hoc power analysis was conducted using an application for simple and complex mediation models, based on Monte Carlo simulations and bootstrapped confidence intervals for the indirect effect^50^. This method calculates power estimates by dividing the number of replications in which the indirect effect of interest is significantly different from zero by the total number of replications.

## Results

### Description of the sample

The total sample consisted of *N* = 94, after excluding 29 participants whose scores on at least one of the variables exceeded the pre-determined Cook’s distance threshold of .03. Participants’ ages ranged from 19 to 74 years (*M* = 43.23, *SD* = 14.02). Five participants were older than the initial exclusion criterion of 65 years, as this criterion was retroactively removed in the YING study. Sixty participants (64%) identified as male, 33 as female, and one as non-binary. At the time of participation, the participants had been diagnosed with an SSD for an average of 13.96 years (*SD* = 11.07; range: 0–43). Eighty-two participants held German citizenship, four Turkish citizenship, three Vietnamese citizenship, and five held other nationalities. A total of 58 participants (62%) were recruited from inpatient care and 36 (38%) from outpatient services. Further sample characteristics are presented in Table 1, while descriptive statistics of the questionnaire scores are provided in Table 2. The mean DASS-21 score for the depression subscale fell within the normal range (0–9) for both clinical and nonclinical samples, as outlined in the DASS-21 manual^51^. Compared to the average PANSS scores reported in the original publication by Kay et al.^42^, this sample exhibited slightly higher scores on the negative symptom scale (*M*_current sample_ = 22.24 vs. *M*_Kay et al., 1987_ = 21.01) and similar scores on the positive symptom scale (*M*_current sample_ = 17.81 vs. *M*_Kay et al., 1987_ = 18.20).

**Table 1:** Sociodemographic characteristics of the sample (*N* = 94). *SD* Standard deviation.

Variables	*n* (%)* |* mean (*SD*)/range
Age (years)	43.23 (14.02)/19–74
Diagnosis	
F20	73 (77.66%)
F21	1 (1.06%)
F22	1 (1.06%)
F23	3 (3.19%)
F25	15 (15.96%)
F29	1 (1.06%)
Recruitment site	
Outpatient	36 (38.30%)
Inpatient	58 (61.70%)
Duration of disease (years)	13.96 (11.07)/0–43
Gender	
Female	33 (35.12%)
Male	60 (63.83%)
Diverse	1 (1.06%)
Nationality	
German	82 (87.23%)
Turkish	4 (4.26%)
Vietnamese	3 (3.19%)
Other	5 (5.32%)
Marital status	
Unmarried	68 (72.34%)
Married	8 (8.51%)
Divorced	17 (18.09%)
Widowed	1 (1.06%)
Housing situation	
Private flat	78 (82.98%)
Shared flat	6 (6.38%)
Assisted living	10 (10.64%)
Children	
Yes	25 (26.60%)
No	69 (73.40%)
Educational level	
No school-leaving certificate	3 (3.19%)
Primary school	12 (12.77%)
Secondary school	24 (25.53%)
A-levels	19 (20.21%)
Technical college	4 (4.26%)
Apprenticeship	12 (12.77%)
Studied	20 (21.28%)
Work situation	
Unemployed	29 (31.18%)
Retired	34 (36.56%)
Student/apprentice	8 (8.60%)
Self-employed	3 (3.23%)
Employed	14 (15.05%)
Other	5 (5.38%)

**Table 2:** Descriptive statistics of the sample (*N* = 94).

Variables	Min–Max	Means in current study; *M* (*SD*)	Comparable study population; *M* (*SD*)
SMQ	25–75	46.64 (10.81)	47.78 (13.99)^a^
PANSS—positive scale	7–30	17.81 (53.31)	16.35 (4.52)^a^
PANSS—negative scale	11–34	22.24 (5.56)	19.78 (4.83)^a^
DASS-21—depression	0–18	7.01 (4.68)	8.30 (5.55)^b^
CFQ	8–42	26.44 (7.98)	25.85 (10.45)^a^

^a^Böge et al. ^25^, ^b^Bergmann et al. ^21^. *M* mean, *SD* standard deviation, *SMQ* Southampton Mindfulness Questionnaire, *PANSS* Positive and Negative Syndrome Scale, *DASS-21* Depression, Anxiety and Stress Scale—21 Items, *CFQ* Cognitive Fusion Questionnaire.

### Zero-order correlations

As predicted in Hypothesis 1, a small-to-moderate negative zero-order correlation was found between mindfulness, as measured by the SMQ, and the PANSS positive scale (*r* =− .24, *p* < .05), as well as the depression subscale of the DASS-21 (*r* = − .26, *p* < .05). Among the SMQ subscales, only *“Absence of Aversion”* exhibited a significant moderate-to-strong negative correlation with the PANSS positive scale (*r* = − .43, *p* < .01), while all subscales except “*Non-Judgment”* significantly correlated with the depression subscale of the DASS-21 (see Table 3).

**Table 3:** Pearson correlations *r* (*N* = 94). *p < .05, **p < .01.

Variables	1	2	3	4	5	6	7	8	9
1 SMQ total	–								
2 SMQ mindful observation	.78**	–							
3 SMQ letting go	.79**	.50**	–						
4 SMQ absence of aversion	.61**	.30**	.28**	–					
5 SMQ non-judgment	.67**	.40**	.44**	.12	–				
6 PANSS—positive scale	− .24*	− .20	− .12	− .43**	.11	–			
7 PANSS—negative scale	− .09	− .07	− .04	− .06	− .09	.40**	–		
8 DASS-21—depression scale	− .26*	− .22*	− .21*	− .16*	− .13	.16	.24*	–	
9 CFQ	− .41**	− .28**	− .43**	− .23*	− .24*	.25*	.29**	.61**	–

*SMQ* Southampton Mindfulness Questionnaire, *PANSS* Positive and Negative Syndrome Scale, *DASS-21* Depression, Anxiety and Stress Scale, *CFQ* Cognitive Fusion Questionnaire.

The correlation between mindfulness and the PANSS negative scale was negative but not significant (*r* = − .09, *p* = .39). Since mediation analyses do not require evidence of simple associations between the IV and DV as a precondition^48,52^, Hypothesis 3b was not rejected, and mediation analysis of the effect of SMQ total scores on PANSS negative scores, with CFQ scores as the mediating variable, was still conducted.

As predicted in Hypothesis 2, the SMQ correlated positively with PF, as indicated by the significant moderate-to-strong negative correlation with the CFQ scores (*r* = − .41, *p* < .01), considering that CF is a process that negatively impacts PF. For a more detailed overview, see Table 3.

### Process analysis

The recruitment site, whether from the in- or outpatient unit at Charité, was included as a covariate in all PROCESS models, following the studies by Bergmann et al.^21^ and Böge et al.^25^. Correlations between the primary outcome variables and recruitment sites are presented in Table 4. Participants from the inpatient ward exhibited significantly higher scores on the CFQ and PANSS positive scale. The detailed results, including *t*- and *p*-values are provided in Table 5.

**Table 4:** Correlations with the covariable recruitment site (*N* = 94). *p < .05, **p < .01.

	SMQ total	PANSS PS	PANSS NS	DASS-21 DS	CFQ
Recruitment site	.13	− .27**	− .20	− .19	− .21*

*SMQ* Southampton Mindfulness Questionnaire, *PANSS* Positive and Negative Syndrome Scale, *PS* Positive Scale, *NS* Negative Scale, *DASS-21 DS* Depression Scale of the Depression, Anxiety and Stress Scale—21 Items, *CFQ* Cognitive Fusion Questionnaire.

**Table 5:** Mean differences in recruitment site (*N* = 94). Patients from the day clinic (*n* = 3) were counted as inpatients.

	Inpatient ward (*n* = 58) *M* (*SD*)	Outpatient facility (*n* = 36) *M* (*SD*)	*t*-test
SMQ total	45.55 (8.95)	48.39 (13.21)	*t* = − 1.24, *p* = .22
CFQ	27.74 (7.56)	24.33 (8.30)	*t* = 2.78, *p* < .05
DASS-21 depression scale	7.71 (4.76)	5.89 (4.37)	*t* = 1.86, *p* = .07
PANSS positive scale	18.91 (4.81)	16.03 (5.64)	*t* = 2.64, *p* < .01
PANSS negative scale	23.12 (5.76)	20.83 (4.99)	*t* = 1.97, *p* = .05

*SMQ* Southampton Mindfulness Questionnaire, *CFQ* Cognitive Fusion Questionnaire, *DASS-21* Depression, Anxiety and Stress Scale—21 Items, *PANSS* Positive and Negative Syndrome Scale.

#### Model A (positive symptom severity)

**Fig. 3:**
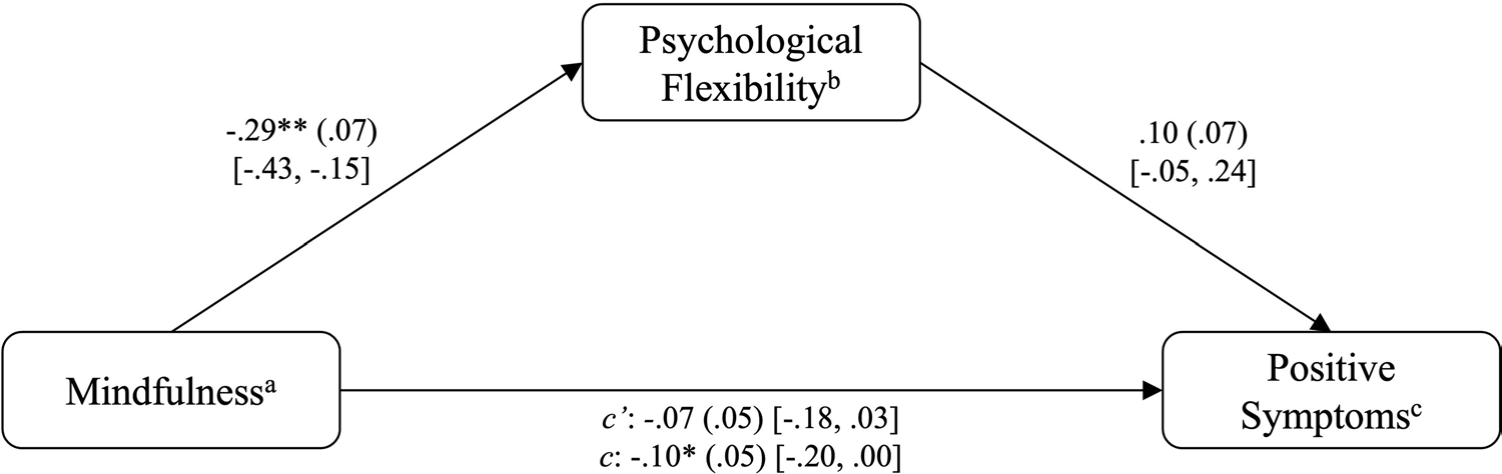
PROCESS outcomes including positive symptoms. *p < .05, **p < .01. ^a^Assessed by the Southampton Mindfulness Questionnaire (SMQ). ^b^Assessed by the Cognitive Fusion Questionnaire (CFQ). ^c^Assessed by the Positive Scale of the Positive and Negative Syndrome Scale (PANSS).

After controlling for the recruitment site as a covariate, a significant total effect of mindfulness on positive symptom severity was found, *B* = − .10, *p* < .05. Mindfulness accounted for *R*^*2*^ = .11 of the variance in positive symptoms (*F*(2,91) = 5.72, *p* < .01). When PF was included in the model as a mediator, mindfulness significantly predicted PF, *B* = − .29, *p* < .01. However, PF did not significantly predict positive symptom severity, *B* = .10, *p* = .18. The model including PF was significant, *F*(3,90) = 4.45, *p* < .01, explaining *R*^*2*^ = .13 of the variance in positive symptoms. The indirect effect of the complete mediation model (*ab* = − .03) was not significant, as the 95% bias-corrected confidence interval based on 10,000 bootstrap samples included zero [95% CI − 0.07, 0.01]. The results are visualized in Fig. 3. A post hoc Monte Carlo power analysis revealed a power of .38, indicating that the indirect effect significantly differed from zero in 38% of the bootstrap replications.

#### Model B (negative symptom severity)

**Fig. 4:**
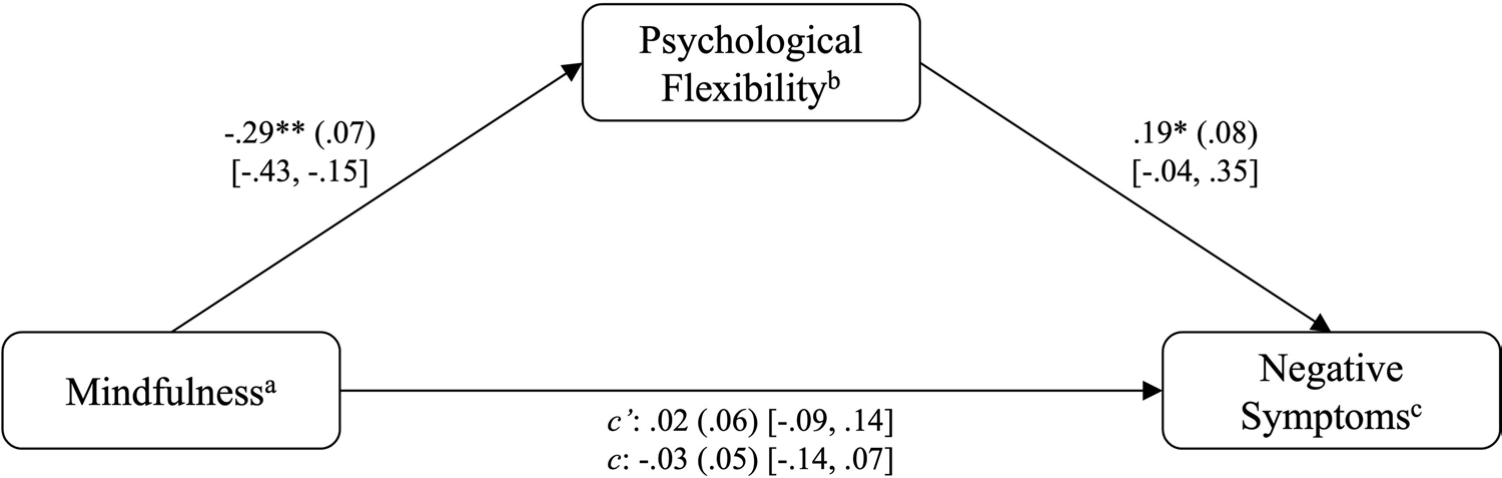
PROCESS outcomes including Negative Symptoms (Model B). *p < .05, **p < .01. ^a^Assessed by the Southampton Mindfulness Questionnaire (SMQ). ^b^Assessed by the Cognitive Fusion Questionnaire (CFQ). ^c^Assessed by the Negative Scale of the Positive and Negative Syndrome Scale (PANSS).

There was no significant total effect of mindfulness on the severity of negative symptoms, *B* = − .03, *p* = .54. Mindfulness accounted for *R*^*2*^ = 0.04 of the variance of negative symptoms (*F*(2,91) = 2.12, *p* = .13). As outlined in prior methodological work, a significant total effect of the IV on the DV is not a prerequisite for mediation, as the indirect effect represents the pure mediation effect^53,54^. After including PF as a mediator in the model, mindfulness significantly predicted PF, *B* = − .29, *p* < .01, which in turn significantly predicted the severity of negative symptoms, *B* = .19, *p* < .05. The model including PF was significant, *F*(3,90) = 3.57, *p* < .05, and explained *R*^*2*^ = .11 of the variance in negative symptoms. Based on the significance of paths a and b, mediation is established^55^. Furthermore, the 95% bias-corrected confidence interval of the indirect effect (*ab* = − .06) did not include zero [95% CI − 0.11, − 0.01], indicating that PF fully mediates the relationship between mindfulness and negative symptom severity. These results are visualized in Fig. 4. A post hoc Monte Carlo power analysis revealed a power of .78 for the indirect effect, indicating that the indirect effect was significantly different from zero in 78% of the bootstrap replications.

#### Model C (depressive symptom severity)

**Fig. 5:**
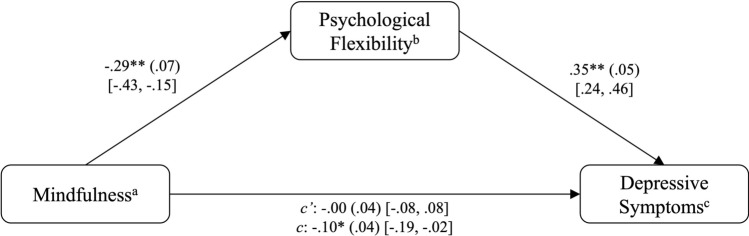
PROCESS outcomes including depressive symptoms (Model C). *p < .05, **p < .01. ^a^Assessed by the Southampton Mindfulness Questionnaire (SMQ). ^b^Assessed by the Cognitive Fusion Questionnaire (CFQ). ^c^Assessed by the depression subscale of the Depression Anxiety Stress Scale (DASS-21).

An effect of mindfulness on depressive symptoms in the absence of PF was observed, *B* = − .10, *p* < .05. Mindfulness accounted for *R*^*2*^ = .09 of the variance in depressive symptoms (*F*(2,91) = 4.56, *p* < .05). After including PF as a mediator in the model, mindfulness significantly predicted the mediator, *B* = − .29, *p* < .01, which in turn significantly predicted depressive symptoms, *B* = .35, *p* < .01. The model that included PF was significant, *F*(3,90) = 17.97, *p* < .01, explaining *R*^*2*^ = .37 of the variance in depressive symptoms. The direct effect of mindfulness on depressive symptoms was no longer significant once the mediator was included, indicating that PF fully mediates the relationship between mindfulness and depressive symptoms, with an indirect effect of *ab* = − 0.10 [95% CI − 0.17, − 0.04]. These results are visualized in Fig. 5. A post hoc Monte Carlo power analysis revealed a high power of .99 for the indirect effect, indicating that the indirect effect was significantly different from zero in 99% of the bootstrap replications.

## Discussion

The present study aimed to explore the relationship between mindfulness and the severity of positive, negative, and depressive symptoms in persons with SSD, with PF as a potential mediator in a cross-sectional design. The first hypothesis proposed that mindfulness would be negatively correlated with the severity of positive (H1a), negative (H1b), and depressive symptoms (H1c). The second hypothesis posited a positive correlation between mindfulness and PF (H2). Finally, it was hypothesized that PF would mediate the relationship between mindfulness and the severity of positive (H3a), negative (H3b), and depressive symptoms (H3c).

In support of the first hypothesis, both positive and depressive symptoms were found to be significantly negatively correlated with mindfulness. The findings regarding positive symptoms align with those of Böge et al.^25^, demonstrating a significant correlation between mindfulness and positive, but not negative, symptoms. Mindfulness practice, which can induce positive emotional experiences such as feelings of calmness and peacefulness^56^, may help reduce the exacerbation of positive symptoms caused by emotional distress, particularly anxiety^57^. Additionally, enhanced metacognitive skills, such as improved interoceptive awareness, may diminish the belief in the negative consequences of psychotic symptoms^58^, thereby mitigating the worsening of positive symptoms. The fact that the mindfulness subscale *“Absence of Aversion”* showed the strongest correlation with positive symptoms (*r* = − .43, *p* < .01) underscores the importance of accepting experiences such as acoustic hallucinations or delusional beliefs without reacting with anger, avoidance, or distraction. This acceptance may reduce symptom-related distress and, consequently, the triggering of these symptoms. Therefore, mindfulness interventions, such as meditation focused on accepting present symptoms, may be particularly effective in targeting positive symptoms.

The negative relationship between mindfulness and depressive symptoms observed in this study is consistent with previous research on persons with SSD^21,25^. This finding also aligns with two recent meta-analyses, which demonstrated that mindfulness- and acceptance-based interventions for persons with psychosis have the most substantial effects on depressive symptoms^10,13^. In the current study, “*Mindful Observation*”, the ability to notice thoughts and feeling without reacting to them, and *“Letting Go”*, the ability to accept challenging cognitions without ruminating or worrying, were most strongly associated with reduced depressive symptoms in SSD. This corresponds with findings that rumination—characterised by repetitive thoughts about current concerns and negative experiences^59^—may represent the counterpart to awareness^60^, particularly *“Mindfulness Observation”* and *“Letting Go”,* and is a primary factor contributing to depression^61,62^. Given that both international (NICE) and national guidelines (S3) now recommend MBIs for the treatment of depression^63,64^, integrating these approaches into the treatment of SSD, where up to 50% of individuals suffer from comorbid depression^65^, could be a promising step towards more effective overall treatment.

In the present study, negative symptoms did not show a significant correlation with mindfulness, consistent with the findings of Böge et al.^25^. Positive symptoms may have a more direct relationship with the concept of mindfulness, whereas the influence of mindfulness on negative symptoms, as indicated in some meta-analyses [e.g., 13,14], may be mediated by other factors. Besides PF, mechanisms such as positive reappraisal^66^, stigma resistance^67^, increased positive emotions and anticipatory pleasure^68^, improved metacognition^69^, or a reduction in cognitive and emotional reactivity and repetitive negative thinking^70^ may mediate the positive effect of mindfulness on negative symptoms. These potential mechanisms should be explored in future studies.

The second hypothesis, which proposed a positive association between mindfulness and PF, was supported by the moderate negative correlation observed between mindfulness and CF, an inverse process of PF. This finding aligns with results from studies conducted in various populations^25,35,37^, as well as with the theoretical background of ACT. In ACT, acceptance and mindfulness processes are employed to cultivate PF, a broad, higher-level construct encompassing six interconnected processes^71^. Notably, four of these processes, namely contact with the present moment, accepting, cognitive defusion, and the conceptualized self, are integral to mindfulness and acceptance practices^30^, highlighting the strong theoretical connection between these two constructs. Rather than conflating thoughts and emotions with reality and allowing them to automatically guide behaviour, a more mindful approach may enable individuals to respond more flexibly and with less automaticity to various thoughts and emotions.

The findings related to the third hypothesis demonstrated that PF mediates the relationship between mindfulness and both negative symptoms and depressive symptoms, but not positive symptoms, in persons with SSD. This outcome aligns with previous research, which suggests PF is an essential mechanisms of change in acceptance- and mindfulness-based approaches^30,72^. Although negative and depressive symptoms are distinct concepts, they share common domains such as anhedonia, avolition, and anergia^73,74^. This underscores the importance of MBIs as transdiagnostic approaches that transcend traditional diagnostic boundaries, opening new avenues for understanding mental well-being and its enhancement through targeted, symptom-cluster-specific interventions.

PF may enhance emotion regulation and foster positive feelings toward others, potentially alleviating symptoms of anhedonia^57^. This could explain why PF mediates the effect of mindfulness on negative and depressive symptoms, but not on positive symptoms. The significant impact of PF on depressive symptoms is further highlighted by the large proportion of variance it explains. Several other studies have also demonstrated that PF contributes more significantly to differences in depressive symptoms than mindfulness alone^36,37,75^. The mediating role of PF between mindfulness and depressive symptoms is additionally supported by longitudinal data^76^, indicating causal directions.

In negative and depressive symptoms occurring in the context of SSD, where also internalized stigma and shame are especially prevalent^77^, the outcomes of this study indicate that focusing on cognitive defusion and PF may enhance the effectiveness of mindfulness-based therapies. However, this hypothesis needs to be confirmed in future trials using randomized-controlled designs. This is particularly relevant given that existing pharmacological treatments and psychological interventions for SSD have shown limited clinical significance for negative symptoms^78^. These symptoms significantly contribute to poor functional outcomes and long-term disability in this population^79,80^. The results of the current study suggest that MBIs could be a promising treatment option for targeting negative and depressive symptoms in SSD, aligning with previous findings on the effectiveness of MBIs (e.g.^81^). Early intervention is crucial, as these symptoms are generally associated with poorer clinical outcomes and an increased risk of suicide^18,20^. Moreover, the recent finding that augmenting mindfulness-based group therapy with oxytocin leads to a greater reduction in negative symptoms compared to a control group receiving mindfulness-based therapy and a placebo^82^ suggests that the potential additive effects of oxytocin on depressive symptoms in MBIs for persons with SSD should be further explored in future studies.

Regarding positive symptoms, different mechanisms of change may explain the impact of mindfulness, as shown by some meta-analyses^11,12,15^. PF is a construct that encompasses six processes, of which five were not considered in this study (i.e., acceptance, being present, values, committed action, and the conceptualized self). It is possible that positive symptoms are alleviated by fostering a present-focused or accepting attitude, rather than by detaching from the literal content of thoughts, an ability that positive symptoms themselves may hinder. Therefore, future studies are needed to investigate the specific mechanisms of change concerning positive symptoms. This will aid in improving MBIs, refining treatment manuals, and facilitating a better selection of patients, depending on their symptoms, who may benefit from mindfulness-work. This study contributes further evidence supporting a potential mechanism of action for the effects of mindfulness on negative and depressive symptoms in SSD, utilizing a diverse sample that includes both inpatients and outpatients, as well as the PANSS as a rater-based instrument in comparison to the Self-Evaluation of Negative Symptoms Scale (SNS) used by Böge et al.^25^.

Nevertheless, these findings should be considered in light of some limitations. Although the sample was larger and more diverse than in previous studies, it was still insufficient to provide adequate power for Model A. The limited statistical power increases the likelihood of Type II errors, whereby true effects may remain undetected despite their presence in this model. Additionally, the cross-sectional design of the study precludes any causal inferences. The directionality of the associations remains unclear, as no temporal precedence was established, and the positions of the variables in the mediation models could potentially be interchangeable. For instance, some findings suggest that CF may predict next-day mindful awareness^34^, while others indicate that mindfulness could act as a mediator between PF and depressive symptoms^83^. Additional mediation models incorporating alternative mediators, such as those mentioned above (e.g., positive reappraisal^66^ or stigma resistance^67^), may offer a more nuanced and precise understanding of the underlying mechanisms and should be compared to the current model in future studies for further validation. Furthermore, although recruitment site was included as a covariate in the statistical analysis, similar to the studies by Bergmann et al.^21^ and Böge et al.^25^, the influence of additional factors such as medication regimens, understanding of the concept of mindfulness, and prior experiences with mindfulness should be examined in future longitudinal trials.

Moreover, the sample may not comprise the diversity in clinical pictures displayed in SSD, as the majority of patients were diagnosed with an ICD-10 F20.x diagnosis (77%). Additionally, the sample predominantly represents chronic stages of psychosis, with a mean illness duration of 13 years. This stage of illness is currently a key focus in research on the effects of mindfulness in SSD^84^. Given that mindfulness, PF, and symptom severity may interact differently in different stages of psychosis, future research should explore these interactions across different subtypes and stages of SSD. Recruitment from clinical settings may introduce bias by excluding persons with milder or untreated psychotic disorders who are not currently receiving care. Additionally, the sample may be skewed toward more stable, motivated patients, as well as those who are more proactive, adherent, or optimistic about treatment, which could lead to self-selection bias. Nonetheless, our sample included both inpatients and outpatients—two groups that may differ in terms of symptom stability and overall psychological functioning, thereby enhancing the generalizability of our findings. In line with this, outpatients (38%) in our sample exhibited significantly greater psychological flexibility and reported lower levels of positive symptoms compared to inpatients. However, no significant group differences emerged for negative symptoms, depressive symptoms, or mindfulness.

Furthermore, mindfulness, PF, and depressive symptom severity were exclusively assessed using self-rated questionnaires. Given the potential discrepancies between individuals’ perceived mindfulness and their actual mindfulness level^85^, which also applies to depressive symptoms and PF, future studies should consider adopting a mixed-methods approach including interviews, self-rating, as well as rater-based instruments. Regarding the rater-based instrument used in this study, it is important to note that interrater-reliability was not assessed, as samples stem from three different studies. This may have contributed to the variance observed in symptom scores.

Moreover, an overlapping, dimensional model of negative, positive, and depressive symptoms in SSD has been proposed^74^, suggesting that future research should examine not only additional mechanisms of mindfulness but also their specific effects on individual symptoms rather than broad symptom categories. This approach could help identify potential effects of mindfulness that were not captured in the current study.

## Conclusion

This study is the first to demonstrate the mediating role of PF in the relationship between mindfulness and depressive symptoms in persons with SSD, while also replicating previous findings on the associations between mindfulness, PF, and negative symptoms. Patients primarily presenting with negative and depressive symptoms may benefit from MBIs that emphasize PF-enhancing techniques, such as cognitive defusion (i.e., viewing thoughts as thoughts rather than absolute truths) or the cultivation of an observer self. In contrast, mindfulness practices targeting alternative mechanisms may be particularly beneficial for persons whose symptomatology is dominated by positive symptoms. Clinicians may consider assessing individual symptom profiles (e.g., depressive vs. positive symptoms) to guide the selection and emphasis of specific MBI components. These potential clinical implications underscore the need for further research to clarify the mechanisms through which mindfulness exerts its effects. Overall, the present findings contribute to refining MBIs and improving their effectiveness for persons with SSD, a population that continues to face substantial barriers to modern, evidence-based treatment.

## Data Availability

The data supporting the findings of this study are available from the corresponding author upon reasonable request.

## References

[1] Kabat-Zinn, J. Mindfulness-based interventions in context: Past, present, and future. *Clin. Psychol. Sci. Pract.* **10**, 144–156. 10.1093/clipsy.bpg016 (2003).

[2] Hilton, L. et al. Mindfulness meditation for chronic pain: Systematic review and meta-analysis. *Ann. Behav. Med.* **51**, 199–213. 10.1007/s12160-016-9844-2 (2017). 27658913 10.1007/s12160-016-9844-2 PMC5368208

[3] Veehof, M. M., Trompetter, H. R., Bohlmeijer, E. T. & Schreurs, K. M. G. Acceptance- and mindfulness-based interventions for the treatment of chronic pain: A meta-analytic review. *Cogn. Behav. Ther.* **45**, 5–31. 10.1080/16506073.2015.1098724 (2016). 26818413 10.1080/16506073.2015.1098724

[4] Li, W., Howard, M. O., Garland, E. L., McGovern, P. & Lazar, M. Mindfulness treatment for substance misuse: A systematic review and meta-analysis. *J. Subst. Abuse Treat.* **75**, 62–96. 10.1016/j.jsat.2017.01.008 (2017). 28153483 10.1016/j.jsat.2017.01.008

[5] Goldberg, S. B. et al. Mindfulness-based cognitive therapy for the treatment of current depressive symptoms: A meta-analysis. *Cogn. Behav. Ther.* **48**, 445–462. 10.1080/16506073.2018.1556330 (2019). 30732534 10.1080/16506073.2018.1556330 PMC6687569

[6] Breedvelt, J. J. F. et al. The effects of meditation, yoga, and mindfulness on depression, anxiety, and stress in tertiary education students: A meta-analysis. *Front. Psychiatry* **10**, 193. 10.3389/fpsyt.2019.00193 (2019). 31068842 10.3389/fpsyt.2019.00193 PMC6491852

[7] Dyga, K. & Stupak, R. Meditation and psychosis. A trigger or a cure?. *Arch. Psychiatry Psychother.* **17**, 48–58. 10.12740/APP/58976 (2015).

[8] Böge, K., Thomas, N. & Jacobsen, P. Is mindfulness for psychosis harmful? Deconstructing a myth. *Br. J. Psychiatry* **218**, 71–72. 10.1192/bjp.2020.165 (2021). 32998787 10.1192/bjp.2020.165

[9] Jacobsen, P., Morris, E., Johns, L. & Hodkinson, K. Mindfulness groups for psychosis: Key issues for implementation on an inpatient unit. *Behav. Cogn. Psychother.* **39**, 349–353. 10.1017/S1352465810000639 (2011). 21092359 10.1017/S1352465810000639

[10] Louise, S., Fitzpatrick, M., Strauss, C., Rossell, S. L. & Thomas, N. Mindfulness- and acceptance-based interventions for psychosis: Our current understanding and a meta-analysis. *Schizophr. Res.* **192**, 57–63. 10.1016/j.schres.2017.05.023 (2018). 28545945 10.1016/j.schres.2017.05.023

[11] Cramer, H., Lauche, R., Haller, H., Langhorst, J. & Dobos, G. Mindfulness- and acceptance-based interventions for psychosis: A systematic review and meta-analysis. *Glob. Adv. Health Med.* **5**, 30–43. 10.7453/gahmj.2015.083 (2016). 26937312 10.7453/gahmj.2015.083 PMC4756771

[12] Hodann-Caudevilla, R. M., Díaz-Silveira, C., Burgos-Julián, F. A. & Santed, M. A. Mindfulness-based interventions for people with schizophrenia: A systematic review and meta-analysis. *Int. J. Environ. Res. Public. Health* **17**, 4690. 10.3390/ijerph17134690 (2020). 32629764 10.3390/ijerph17134690 PMC7369977

[13] Jansen, J. E., Gleeson, J., Bendall, S., Rice, S. & Alvarez-Jimenez, M. Acceptance- and mindfulness-based interventions for persons with psychosis: A systematic review and meta-analysis. *Schizophr. Res.* **215**, 25–37. 10.1016/j.schres.2019.11.016 (2020). 31780349 10.1016/j.schres.2019.11.016

[14] Khoury, B., Lecomte, T., Gaudiano, B. A. & Paquin, K. Mindfulness interventions for psychosis: A meta-analysis. *Schizophr. Res.* **150**, 176–184. 10.1016/j.schres.2013.07.055 (2013). 23954146 10.1016/j.schres.2013.07.055

[15] Yip, A. L. K., Karatzias, T. & Chien, W. T. Mindfulness-based interventions for non-affective psychosis: A comprehensive systematic review and meta-analysis. *Ann. Med.* **54**, 2339–2352. 10.1080/07853890.2022.2108551 (2022). 10.1080/07853890.2022.2108551 PMC9423825 36004784

[16] Meinhart, A., Schmueser, A., Moritz, S. & Böge, K. Effects of mindfulness- and acceptance-based interventions for individuals with schizophrenia spectrum disorders: A systematic meta-review. *Schizophr. Res.* **281**, 91–107. 10.1016/j.schres.2025.03.040 (2025). 40328093 10.1016/j.schres.2025.03.040

[17] Li, W. et al. Prevalence of comorbid depression in schizophrenia: A meta-analysis of observational studies. *J. Affect. Disord.* **273**, 524–531. 10.1016/j.jad.2020.04.056 (2020). 32560949 10.1016/j.jad.2020.04.056

[18] Dutta, R., Murray, R. M., Allardyce, J., Jones, P. B. & Boydell, J. Early risk factors for suicide in an epidemiological first episode psychosis cohort. *Schizophr. Res.* **126**, 11–19. 10.1016/j.schres.2010.11.021 (2011). 21183318 10.1016/j.schres.2010.11.021

[19] Popovic, D. et al. Risk factors for suicide in schizophrenia: Systematic review and clinical recommendations. *Acta Psychiatr. Scand.* **130**, 418–426. 10.1111/acps.12332 (2014). 25230813 10.1111/acps.12332

[20] Upthegrove, R., Marwaha, S. & Birchwood, M. Depression and schizophrenia: Cause, consequence or trans-diagnostic issue?. *Schizophr. Bull.*10.1093/schbul/sbw097 (2016). 10.1093/schbul/sbw097 PMC5605248 27421793

[21] Bergmann, N. et al. The relationship between mindfulness, depression, anxiety, and quality of life in individuals with schizophrenia spectrum disorders. *Front. Psychol.* **12**, 708808. 10.3389/fpsyg.2021.708808 (2021). 34531796 10.3389/fpsyg.2021.708808 PMC8438172

[22] Sabé, M. et al. Mindfulness-based interventions for patients with schizophrenia spectrum disorders: A systematic review of the literature. *Schizophr. Res.* **264**, 191–203. 10.1016/j.schres.2023.12.011 (2024). 38157679 10.1016/j.schres.2023.12.011

[23] Chadwick, P. Mindfulness for psychosis. *Br. J. Psychiatry* **204**, 333–334. 10.1192/bjp.bp.113.136044 (2014). 24785766 10.1192/bjp.bp.113.136044

[24] Shapiro, S. L., Carlson, L. E., Astin, J. A. & Freedman, B. Mechanisms of mindfulness. *J. Clin. Psychol.* **62**, 373–386. 10.1002/jclp.20237 (2006). 16385481 10.1002/jclp.20237

[25] Böge, K. et al. Mindfulness, cognitive fusion, and self-compassion in patients with schizophrenia spectrum disorders—A cross-sectional study. *Front. Psychiatry* **13**, 959467. 10.3389/fpsyt.2022.959467 (2022). 35982935 10.3389/fpsyt.2022.959467 PMC9378854

[26] Kashdan, T. B. & Rottenberg, J. Psychological flexibility as a fundamental aspect of health. *Clin. Psychol. Rev.* **30**, 865–878. 10.1016/j.cpr.2010.03.001 (2010). 21151705 10.1016/j.cpr.2010.03.001 PMC2998793

[27] Böge, K., Hallford, D. J. & Pillny, M. Mindfulness, psychological flexibility and their relationship with psychopathology in persons with schizophrenia-spectrum-disorders and healthy controls—A multicenter cross-sectional study. *Psychiatry Res.* **330**, 115591. 10.1016/j.psychres.2023.115591 (2023). 37979316 10.1016/j.psychres.2023.115591

[28] Cansız, A., Nalbant, A. & Yavuz, K. Investigation of psychological flexibility in patients with schizophrenia. *J. Cogn.-Behav. Psychother. Res.*10.5455/JCBPR.55825 (2019).

[29] Muntean, L. M. et al. The relationship between unconditional self-acceptance and cognitive fusion in psychosis. *Psihiatru.ro* **3**, 26. 10.26416/Psih.62.3.2020.3877 (2020).

[30] Hayes, S. C., Pistorello, J. & Levin, M. E. Acceptance and commitment therapy as a unified model of behavior change. *Couns. Psychol.* **40**, 976–1002. 10.1177/0011000012460836 (2012).

[31] Klimczak, K. S. & Levin, M. E. Acceptance and commitment therapy. In *Reference Module in Neuroscience and Biobehavioral Psychology* B9780323914970001211 (Elsevier, 2022).

[32] Gillanders, D. T. et al. The development and initial validation of the cognitive fusion questionnaire. *Behav. Ther.* **45**, 83–101. 10.1016/j.beth.2013.09.001 (2014). 24411117 10.1016/j.beth.2013.09.001

[33] Bardeen, J. R. & Fergus, T. A. The interactive effect of cognitive fusion and experiential avoidance on anxiety, depression, stress and posttraumatic stress symptoms. *J. Context. Behav. Sci.* **5**, 1–6. 10.1016/j.jcbs.2016.02.002 (2016).

[34] Berghoff, C. R., Ritzert, T. R. & Forsyth, J. P. Value-guided action: Within-day and lagged relations of experiential avoidance, mindful awareness, and cognitive fusion in a non-clinical sample. *J. Context. Behav. Sci.* **10**, 19–23. 10.1016/j.jcbs.2018.07.005 (2018).

[35] Moran, O., Larsson, A. & McHugh, L. Investigating cognitive fusion, mindfulness and experiential avoidance in relation to psychosis-like symptoms in the general population. *J. Context. Behav. Sci.* **21**, 136–143. 10.1016/j.jcbs.2021.06.004 (2021).

[36] Pinto-Gouveia, J., Dinis, A., Gregório, S. & Pinto, A. M. Concurrent effects of different psychological processes in the prediction of depressive symptoms—The role of cognitive fusion. *Curr. Psychol.* **39**, 528–539. 10.1007/s12144-017-9767-5 (2020).

[37] Vancappel, A., Courtois, R., Réveillère, C. & El-Hage, W. Interaction of mediation and moderation effects of positivity, cognitive fusion, brooding and mindfulness. *L’Encéphale* **49**, 227–233. 10.1016/j.encep.2021.12.003 (2023). 35221020 10.1016/j.encep.2021.12.003

[38] García-Gómez, M., Guerra, J., López-Ramos, V. M. & Mestre, J. M. Cognitive fusion mediates the relationship between dispositional mindfulness and negative affects: A study in a sample of Spanish children and adolescent school students. *Int. J. Environ. Res. Public. Health* **16**, 4687. 10.3390/ijerph16234687 (2019). 31775280 10.3390/ijerph16234687 PMC6926870

[39] Pux, S. et al. Cognitive fusion and personality traits in the context of mindfulness: A cross-sectional study. *PLoS ONE* **17**, e0273331. 10.1371/journal.pone.0273331 (2022). 36170277 10.1371/journal.pone.0273331 PMC9518896

[40] Harris, P. A. et al. The REDCap consortium: Building an international community of software platform partners. *J. Biomed. Inform.* **95**, 103208. 10.1016/j.jbi.2019.103208 (2019). 31078660 10.1016/j.jbi.2019.103208 PMC7254481

[41] Böge, K. et al. Validation of the german version of the southampton mindfulness questionnaire (SMQ). *Mindfulness* **11**, 2219–2234. 10.1007/s12671-020-01447-x (2020).

[42] Kay, S. R., Fiszbein, A. & Opler, L. A. The positive and negative syndrome scale (PANSS) for schizophrenia. *Schizophr. Bull.* **13**, 261–276. 10.1093/schbul/13.2.261 (1987). 3616518 10.1093/schbul/13.2.261

[43] Peralta, V. & Cuesta, M. J. Psychometric properties of the positive and negative syndrome scale (PANSS) in schizophrenia. *Psychiatry Res.* **53**, 31–40. 10.1016/0165-1781(94)90093-0 (1994). 7991730 10.1016/0165-1781(94)90093-0

[44] Nilges, P. & Essau, C. DASS. Depressions-Angst-Stress-Skalen—deutschsprachige Kurzfassung (2021). 10.23668/psycharchives.15830

[45] Page, A. C., Hooke, G. R. & Morrison, D. L. Psychometric properties of the depression anxiety stress scales (DASS) in depressed clinical samples. *Br. J. Clin. Psychol.* **46**, 283–297. 10.1348/014466506X158996 (2007). 17697478 10.1348/014466506X158996

[46] China, C., Hansen, L. B., Gillanders, D. T. & Benninghoven, D. Concept and validation of the German version of the cognitive fusion questionnaire (CFQ-D). *J. Context. Behav. Sci.* **9**, 30–35. 10.1016/j.jcbs.2018.06.003 (2018).

[47] R Core Team. *R: A Language and Environment for Statistical Computing*. R Foundation for Statistical Computing. https://www.R-project.org/ (2023).

[48] Hayes, A. F. *Introduction to Mediation, Moderation, and Conditional Process Analysis: A Regression-Based Approach* (The Guilford Press, 2022).

[49] Preacher, K. J., Rucker, D. D. & Hayes, A. F. Addressing moderated mediation hypotheses: Theory, methods, and prescriptions. *Multivar. Behav. Res.* **42**, 185–227. 10.1080/00273170701341316 (2007). 10.1080/00273170701341316 26821081

[50] Schoemann, A. M., Boulton, A. J. & Short, S. D. Determining power and sample size for simple and complex mediation models. *Soc. Psychol. Personal. Sci.* **8**, 379–386. 10.1177/1948550617715068 (2017).

[51] Lovibond, S. H. & Lovibond, P. F. *Manual for the Depression Anxiety Stress Scales* (Psychology Foundation of Australia, 1995).

[52] Bollen, K. A. *Structural Equations with Latent Variables: Bollen/Structural Equations with Latent Variables* (Wiley, 1989).

[53] Rucker, D. D., Preacher, K. J., Tormala, Z. L. & Petty, R. E. Mediation analysis in social psychology: Current Practices and New Recommendations. *Soc. Personal. Psychol. Compass* **5**, 359–371. 10.1111/j.1751-9004.2011.00355.x (2011).

[54] Zhao, X., Lynch, J. G. & Chen, Q. Reconsidering baron and kenny: Myths and truths about mediation analysis. *J. Consum. Res.* **37**, 197–206. 10.1086/651257 (2010).

[55] MacKinnon, D. P. *Introduction to Statistical Mediation Analysis* (Routledge, 2012).

[56] Shaner, L., Kelly, L., Rockwell, D. & Curtis, D. Calm abiding: The lived experience of the practice of long-term meditation. *J. Humanist. Psychol.* **57**, 98–121. 10.1177/0022167815594556 (2017).

[57] Liu, Y., Li, I. & Hsiao, F. Effectiveness of mindfulness-based intervention on psychotic symptoms for patients with schizophrenia: A meta-analysis of randomized controlled trials. *J. Adv. Nurs.* **77**, 2565–2580. 10.1111/jan.14750 (2021). 33450107 10.1111/jan.14750

[58] Chadwick, P. *Person-Based Cognitive Therapy for Distressing Psychosis* (Wiley, 2006).

[59] Raes, F. et al. Repetitive negative thinking outperforms loneliness and lack of social connectedness as a predictor of prospective depressive symptoms in adolescents. *Scand. J. Child Adolesc. Psychiatry Psychol.* **8**, 149–156. 10.21307/sjcapp-2020-015 (2020). 10.21307/sjcapp-2020-015 PMC7863726 33564631

[60] Ludwig, L., Mehl, S., Schlier, B., Krkovic, K. & Lincoln, T. M. Awareness and rumination moderate the affective pathway to paranoia in daily life. *Schizophr. Res.* **216**, 161–167. 10.1016/j.schres.2019.12.007 (2020). 31892492 10.1016/j.schres.2019.12.007

[61] Lam, A. H. Y., Cheung, Y. T. D., Wong, K. H., Leung, S. F. & Chien, W. T. Dispositional mindfulness and psychotic symptoms in schizophrenia spectrum disorders: The mediating roles of rumination and negative emotion. *Neuropsychiatr. Dis. Treat.* **18**, 75–85. 10.2147/NDT.S338133 (2022). 35046658 10.2147/NDT.S338133 PMC8760986

[62] Kovács, L. N. et al. Rumination in major depressive and bipolar disorder—A meta-analysis. *J. Affect. Disord.* **276**, 1131–1141. 10.1016/j.jad.2020.07.131 (2020). 32777651 10.1016/j.jad.2020.07.131

[63] Arbeitsgemeinschaft für Neuropsychopharmakologie und Pharmakopsychiatrie E.V*. Nationale VersorgungsLeitlinie Unipolare Depression - Kurzfassung*. (2022). 10.6101/AZQ/000498

[64] National Institute for Health and Care Excellence (NICE). Depression in adults: treatment and management. (2022). https://www.nice.org.uk/guidance/ng222 35977056

[65] Buckley, P. F., Miller, B. J., Lehrer, D. S. & Castle, D. J. Psychiatric comorbidities and schizophrenia. *Schizophr. Bull.* **35**, 383–402. 10.1093/schbul/sbn135 (2009). 19011234 10.1093/schbul/sbn135 PMC2659306

[66] Garland, E. L., Gaylord, S. A. & Fredrickson, B. L. Positive reappraisal mediates the stress-reductive effects of mindfulness: An upward spiral process. *Mindfulness* **2**, 59–67. 10.1007/s12671-011-0043-8 (2011).

[67] Aliche, C. J., Ifeagwazi, C. M., Nwamarah, J. U., Okechukwu, F. O. & Ngwu, E. C. Mediating roles of positive reappraisal and stigma resistance in the relationship between mindfulness and quality of life among stable schizophrenia patients. *Curr. Psychol.*10.1007/s12144-023-04563-8 (2023).

[68] Johnson, D. P. et al. A pilot study of loving-kindness meditation for the negative symptoms of schizophrenia. *Schizophr. Res.* **129**, 137–140. 10.1016/j.schres.2011.02.015 (2011). 21385664 10.1016/j.schres.2011.02.015

[69] Swanson, L., Griffiths, H., Moritz, S. & Cervenka, S. Metacognitive training for negative symptoms: Support for the cognitive model. *Clin. Psychol. Psychother.* **30**, 486–490. 10.1002/cpp.2809 (2023). 36494180 10.1002/cpp.2809

[70] Gu, J., Strauss, C., Bond, R. & Cavanagh, K. How do mindfulness-based cognitive therapy and mindfulness-based stress reduction improve mental health and wellbeing? A systematic review and meta-analysis of mediation studies. *Clin. Psychol. Rev.* **37**, 1–12. 10.1016/j.cpr.2015.01.006 (2015). 25689576 10.1016/j.cpr.2015.01.006

[71] Gloster, A. T., Klotsche, J., Chaker, S., Hummel, K. V. & Hoyer, J. Assessing psychological flexibility: What does it add above and beyond existing constructs?. *Psychol. Assess.* **23**, 970–982. 10.1037/a0024135 (2011). 21767025 10.1037/a0024135

[72] Lee, J. W. & Park, H. S. Development and effects of an acceptance commitment-based cognitive behavioral program for patients with schizophrenia. *J. Korean Acad. Psychiatr. Ment. Health Nurs.* **27**, 342. 10.12934/jkpmhn.2018.27.4.342 (2018).

[73] Edwards, C. J., Garety, P. & Hardy, A. The relationship between depressive symptoms and negative symptoms in people with non-affective psychosis: A meta-analysis. *Psychol. Med.* **49**, 2486–2498. 10.1017/S0033291719002381 (2019). 31530319 10.1017/S0033291719002381

[74] Krynicki, C. R., Upthegrove, R., Deakin, J. F. W. & Barnes, T. R. E. The relationship between negative symptoms and depression in schizophrenia: A systematic review. *Acta Psychiatr. Scand.* **137**, 380–390. 10.1111/acps.12873 (2018). 29532909 10.1111/acps.12873

[75] White, R. G. et al. Depression and anxiety following psychosis: Associations with mindfulness and psychological flexibility. *Behav. Cogn. Psychother.* **41**, 34–51. 10.1017/S1352465812000239 (2013). 22578715 10.1017/S1352465812000239

[76] Nitzan-Assayag, Y., Aderka, I. M. & Bernstein, A. Dispositional mindfulness in trauma recovery: Prospective relations and mediating mechanisms. *J. Anxiety Disord.* **36**, 25–32. 10.1016/j.janxdis.2015.07.008 (2015). 26401969 10.1016/j.janxdis.2015.07.008

[77] Gumley, A., Braehler, C., Laithwaite, H., MacBeth, A. & Gilbert, P. A compassion focused model of recovery after psychosis. *Int. J. Cogn. Ther.* **3**, 186–201. 10.1521/ijct.2010.3.2.186 (2010).

[78] Fusar-Poli, P. et al. Treatments of negative symptoms in schizophrenia: Meta-analysis of 168 randomized placebo-controlled trials. *Schizophr. Bull.* **41**, 892–899. 10.1093/schbul/sbu170 (2015). 25528757 10.1093/schbul/sbu170 PMC4466178

[79] Correll, C. U. & Schooler, N. R. Negative symptoms in schizophrenia: A review and clinical guide for recognition, assessment, and treatment. *Neuropsychiatr. Dis. Treat.* **16**, 519–534. 10.2147/NDT.S225643 (2020). 32110026 10.2147/NDT.S225643 PMC7041437

[80] Rabinowitz, J. et al. Negative symptoms have greater impact on functioning than positive symptoms in schizophrenia: Analysis of CATIE data. *Schizophr. Res.* **137**, 147–150. 10.1016/j.schres.2012.01.015 (2012). 22316568 10.1016/j.schres.2012.01.015

[81] Böge, K. et al. Mindfulness-based group therapy for in-patients with schizophrenia spectrum disorders—Feasibility, acceptability, and preliminary outcomes of a rater-blinded randomized controlled trial. *Schizophr. Res.* **228**, 134–144. 10.1016/j.schres.2020.12.008 (2021). 33434727 10.1016/j.schres.2020.12.008

[82] Zierhut, M. et al. The combination of oxytocin and mindfulness-based group therapy for empathy and negative symptoms in schizophrenia spectrum disorders—A double-blinded, randomized, placebo-controlled pilot study. *J. Psychiatr. Res.* **171**, 222–229. 10.1016/j.jpsychires.2024.01.014 (2024). 38309212 10.1016/j.jpsychires.2024.01.014

[83] Curtiss, J. & Klemanski, D. H. Teasing apart low mindfulness: Differentiating deficits in mindfulness and in psychological flexibility in predicting symptoms of generalized anxiety disorder and depression. *J. Affect. Disord.* **166**, 41–47. 10.1016/j.jad.2014.04.062 (2014). 25012409 10.1016/j.jad.2014.04.062

[84] Reich, D., Evans, S., Nelson, B., Hickey, T. & O’Shea, M. Evidence map of mindfulness for stages of psychosis: State of the literature and implications for future research. *Mindfulness* **12**, 1860–1877. 10.1007/s12671-021-01611-x (2021).

[85] Grossman, P. On measuring mindfulness in psychosomatic and psychological research. *J. Psychosom. Res.* **64**, 405–408. 10.1016/j.jpsychores.2008.02.001 (2008). 18374739 10.1016/j.jpsychores.2008.02.001

